# Effect of Osimertinib in Combination With Chemotherapy and Bevacizumab for Untreated Epidermal Growth Factor Receptor–Mutated Advanced Non-Small-Cell Lung Cancer: Case Report

**DOI:** 10.3389/fphar.2021.679667

**Published:** 2021-07-22

**Authors:** Haifeng Qin, Fang Wang, Zhen Zeng, Suting Jia, Yuan Liu, Hongjun Gao

**Affiliations:** ^1^ Department of Pulmonary Neoplasm Internal Medicine, Fifth Medical Center of Chinese PLA General Hospital, Beijing, China; ^2^ Department of Internal Medicine of OASIS International Hospital, Beijing, China; ^3^ AstraZeneca Pharmaceutical Limited Company, Beijing, China

**Keywords:** Osimertinib, EGFR, L858R, combined therapy, bevacizumab, NSCLC

## Abstract

**Introduction:** Osimertinib is an oral, third-generation, irreversible epidermal growth factor receptor tyrosine kinase inhibitor (EGFR-TKI) that selectively inhibits both EGFR-TKI–sensitizing and *EGFR* T790M resistance mutations. However, whether patients with EGFR mutations can be treated by osimertinib in combination with conventional therapies, remains unknown.

**Case presentation:** We treated a 67-year-old female diagnosed with non-small-cell lung cancer with an EGFR 21 exon L858R–positive mutation. The patient was treated with 80 mg orally administered osimertinib daily, 830 mg pemetrexed, 120 mg cisplatin, and 500 mg bevacizumab. After two cycles of therapy, the patient’s intrapulmonary lesions shrank from 18 × 24 mm to 16 × 4 mm. Moreover, two cycles of evaluation were PR, and four cycles of confirmation were PR. The patient continued to receive the treatments and tolerated them well.

**Conclusions:** The patient benefited from treatment with osimertinib in combination with chemotherapy and bevacizumab.

## Introduction

Osimertinib is an oral, third-generation, irreversible epidermal growth factor receptor tyrosine kinase inhibitor (EGFR-TKI) that can selectively inhibit both EGFR-TKI–sensitizing and *EGFR* T790M resistance mutations. Currently, the standard treatment options for patients with locally advanced or metastatic non-small-cell lung cancer (NSCLC) harboring EGFR-TKI–sensitizing mutations include first-generation EGFR-TKIs, second-generation EGFR-TKIs, such as gefitinib, erlotinib, and afatinib, and third-generation EGFR-TKIs, such as osimertinib (Soria et al., 2018). However, whether the treatment of patients with an EGFR mutation can be combined with conventional therapies, such as chemotherapy, is unclear. Therefore, this study reported on a case study to explore the effectiveness of osimertinib in combination with chemotherapy and bevacizumab in patients with untreated EGFRm + NSCLC to provide further insight into the treatment of those with EGFR-mutated advanced NSCLC.

## Case Presentation

**FIGURE 1:**
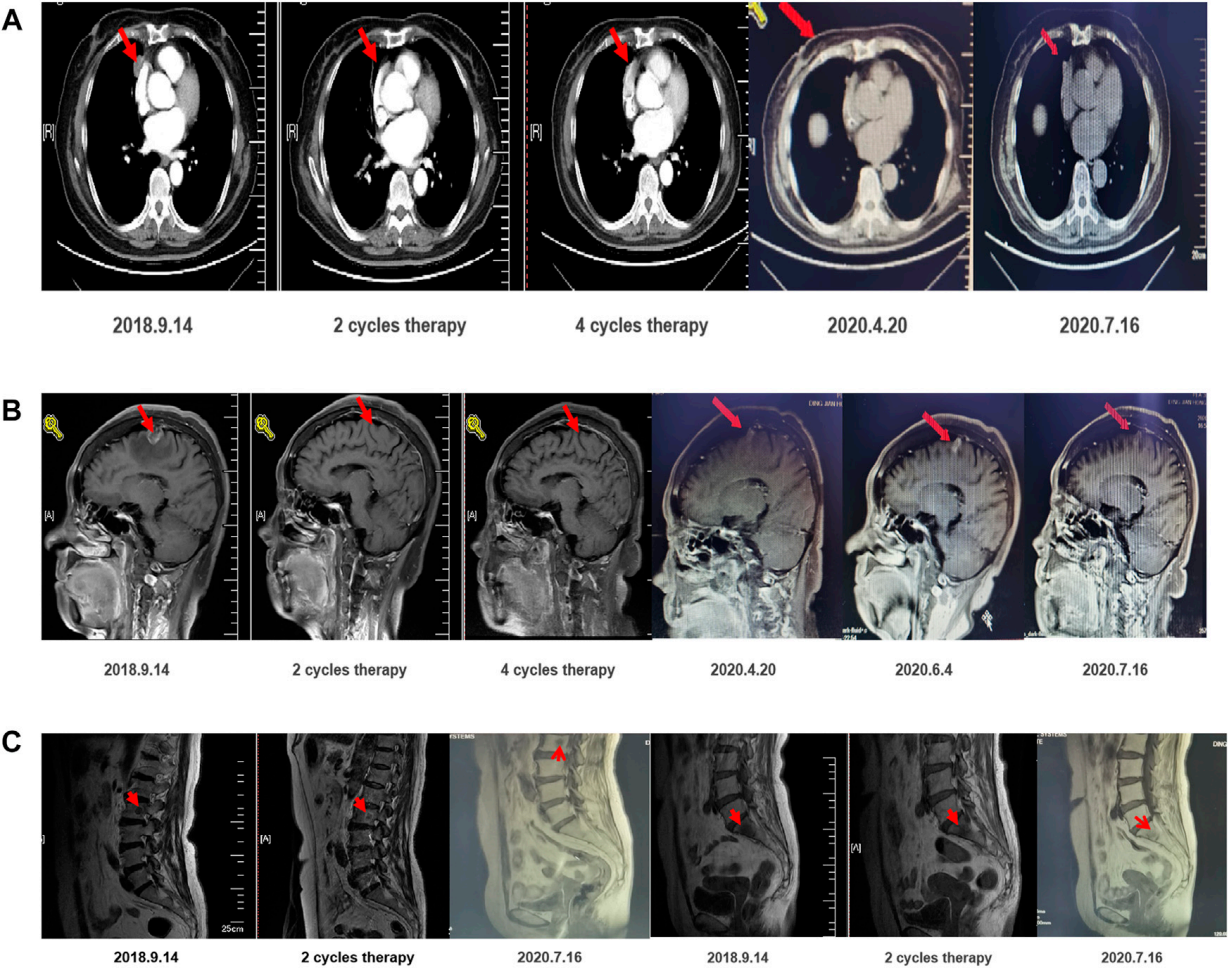
Imaging regression of the lesion demonstrates the effectiveness of the treatment regimen.

A previously healthy woman aged 67 years presented with CEA (34.79 ng/ml) upon a physical examination on August 8, 2018. The patient was diagnosed with adenocarcinoma IV (T2aN2M1a) in the right lung with mediastinal lymph node metastasis, multiple bone metastasis, liver metastasis, and multiple brain metastases by PET-CT on September 7 (Figure 1). The scan demonstrated that the mediastinal metabolic activity of the medial segment of the middle lobe of the right lung was significantly increased. The nodule sizes were around 1.4, 2.4, and 3.3 cm and the SUV_max_ 25.1, indicating lung cancer. There were right mediastinal multiple lymph node metastases, and the multiple radioactive uptakes were increased in the 2R, 4R, and 7 regions. The larger one had a short diameter of 1.5 cm and an SUV_max_ of 17.7. There was metastasis in the liver VIII section, lumbar vertebrae 2 and 5, and sacral bone. The metabolic activity of the above metastatic lesions was significantly increased. A slightly metabolically active nodule in the posterior segment of the upper lobe tip of the right lung was considered possible lung metastasis. On September 13, the immunohistochemical results from the hospital revealed hepatocyte(–), TTF-1(–), napsin A(+), PD-1(–), and PDL1(–). An ultrasound-guided liver biopsy revealed moderately differentiated adenocarcinoma with necrosis, which was consistent with lung origin in combination with the patient’s medical history and immunohistochemistry. The results of the tissue and blood genetic tests indicated an EGFR 21 exon L858R–positive mutation. Moreover, the ECOG PS score was zero.

## Summary of the Relevant Literature

### Flaura Trial

This trial compared osimertinib with standard EGFR-TKIs in patients with previously untreated EGFR mutation–positive advanced NSCLC. The trial found that osimertinib significantly prolonged the PFS benefit and reduced the risk of disease progression. The median overall survival was 38.6 months in the osimertinib group and 31.8 months in the comparator group.

Osimertinib has been shown to benefit from prolonged PFS and OS in both positive and negative subgroups of CNS metastasis (Soria et al., 2018; Ramalingam et al., 2020).

In summary, osimertinib is recommended as a novel first-line standard agent for the treatment of advanced NSCLC patients with EGFR mutations.

### NEJ026 Trial

This trial was a phase III clinical study that compared erlotinib combined with bevacizumab and erlotinib monotherapy in untreated patients with advanced non-squamous NSCLC with EGFR-sensitive mutations. The median PFS of the bevacizumab plus erlotinib (BE) group was longer than that of the erlotinib (E) group.

Adverse events, such as bleeding, proteinuria, and hypertension, occurred significantly higher in the BE group than in the E group. In the E group, five cases of low to moderate pneumonia occurred, while in the BE group, neither pneumonia nor treatment-related deaths occurred (Maemondo et al., 2016; Saito et al., 2019).

The combination of bevacizumab and erlotinib working as combined inhibitors of EGFR-TKIs and VEGF achieved a sustained response and showed good tolerance.

### NEJ009 Trial

This trial was a phase III clinical study that compared the efficacy of gefitinib monotherapy (G) with a combined therapy consisting of gefitinib, carboplatin, and pemetrexed (GCP) in patients with advanced NSCLC along with a primary EGFR-sensitive mutation. The ITT dataset included 344 patients with balanced baseline characteristics. The GCP group demonstrated a better ORR and PFS than the G group, although the PFS2 was not significantly different. The median OS in the GCP group was also significantly longer than that in the G group (Hosomi et al., 2020).

## Suggested Approach to Management

There are several first-line treatment options for EGFR mutations (Saito et al., 2019). Currently, the three generations of EGFR-TKIs on the market can be combined with chemotherapy and anti-angiogenesis therapy. The present research investigated which of these therapies represents the best option.

We weighed the value of the three generations of EGFR-TKIs in patients and found that the efficacy and toxicity profiles strongly favored osimertinib (Hosomi et al., 2020). Furthermore, a phase I trial of osimertinib in combination with bevacizumab found that 76% (13/17) of patients achieved partial or complete remission, with fewer toxic side effects. Furthermore, according to the NEJ026 and NEJ009 trials, the combined therapy showed a better benefit. The overall survival of the patients depended on the outcome of the initial treatment. In short, the higher the initial response rate and the longer the response time, the longer the patients’ lifespan. Therefore, the most effective treatment should be used as the first-line treatment. Our patient had multiple metastatic lesions at the time of initial treatment. Osimertinib combined with chemotherapy and anti-angiogenic therapy was recommended and led to an excellent anti-tumor response (Minari et al., 2016; Batson et al., 2017).

After communication, the patient accepted and was treated with 80 mg orally administered osimertinib daily, 830 mg pemetrexed, 120 mg cisplatin, and 7.5 mg/kg bevacizumab. After two therapy cycles, the intrapulmonary lesions shrank from 18 × 24 mm to 16 × 4 mm. Two cycles of confirmation were PR, and four cycles of confirmation were PR. The patient developed symptoms of two degrees of white blood cell, neutrophil, and platelet reduction following chemotherapy. Thus, corresponding adjuvant therapy was adopted. Due to adverse hematological or gastrointestinal reactions after previous chemotherapy, the systemic chemotherapy regimen was adjusted to the fifth and sixth cycles of pemetrexed combined with bevacizumab. No discomfort was reported after the fifth cycle of chemotherapy, and the hematological toxicity was reduced. Then, from March 3, 2019, to April 28, 2020, the maintenance treatment was osimertinib combined with bevacizumab (7.5 mg/kg), and f cycles of confirmation were PR. On June 4, 2020, the intracerebral lesion increased, and, therefore, the dosage of bevacizumab was adjusted from 7.5 to 15 mg/kg. One month later, the re-examination indicated that the patient was benefiting well from the treatment (Figure 1). The patient continues to receive these treatment options with good tolerance.

## Discussion

This study investigated whether the first-line treatment for patients with EGFR mutations could be combined with conventional therapies, such as anti-angiogenesis and/or chemotherapy. According to the current remission rate, regression depth, and PFS data, the main benefit of combining anti-angiogenesis or chemotherapy is to reduce the tumor load and heterogeneous reserve to extend the remission time and delay the occurrence of drug resistance to extend the PFS (Ballard et al., 2016; Yu, 2017). In addition, combined chemotherapy can also increase the remission population. Despite a gleam of PFS data, the benefit of the OS requires further validation because chemotherapy and anti-angiogenesis lead to drug resistance following conventional treatment options. The backup method of the premise reduces rescue measures and extends the use time of the backup. Considering the cost of treatment and the toxicity, the superposition of treatment should be able to alter the biological behavior of tumors to extend overall survival. Therefore, the OS should be used as the primary endpoint. A growing body of evidence shows that the follow-up treatment of cross does not explain OS, the main reasons for treatment failure, or whether the treatment can significantly change the biological process of tumor growth. However, first-line treatment plays a decisive role in the OS. Currently, only targeted drugs combined with chemotherapy have achieved positive results in phase III studies with OS as the main endpoint (NEJ009), while anti-angiogenesis has not been shown to improve the OS. A recent clinical study showed that the 12-months PFS of the combination of osimertinib and bevacizumab treatment for patients with metastatic EGFR-mutant lung cancer was 76% (95% CI, 65–90%). In our case report, we used osimertinib, bevacizumab, and cisplatin to treat a patient with EGFR-mutant advanced NSCLC, which may provide further insight into the treatment of patients with this disease (Asuda et al., 2017).

In conclusion, the patient with EGFR-mutated advanced NSCLC received combination therapy with good tolerance and benefited from the treatment with osimertinib in combination with chemotherapy and bevacizumab. This case explored the combination therapy model in patients with rapid progress (Seto et al., 2014). Five other patients are currently receiving the same protocol and are showing good benefits. (Feng et al., 2018; Wu, 2019). The greatest advantage of this protocol is that it minimizes the tumor burden in the short term, thus increasing the patients’ overall survival, especially for those with good performance and concomitant mutations. However, the protocol also has some limitations, such as economic burden, off-label use, and a small case sample. In future research, we aim to further explore this protocol in large populations and populations most suited to the combination therapy.

## Data Availability

The original contributions presented in the study are included in the article/supplementary material, further inquiries can be directed to the corresponding author.
